# The Prevalence of Depression, Anxiety, and Stress in Women with Recurrent Pregnancy Loss and Infertility Compared to Normal Controls in Oman: A Prospective, Cross-Sectional Study

**DOI:** 10.3390/healthcare14040453

**Published:** 2026-02-11

**Authors:** Manal Al-Busaidi, Wadha Al-Ghafri, Maryam Al Shukri, Hamed Al-Sinawi, Rahma Al-Ghabshi, Vaidyanathan Gowri

**Affiliations:** 1 Obstetrics and Gynecology Department, University Medical City, Muscat 123, Oman; m.albusaidi3@squ.edu.om (M.A.-B.); wghafri@squ.edu.om (W.A.-G.); mnalshukri@gmail.com (M.A.S.); 2 Behavioral Medicine Department, University Medical City, Muscat 123, Oman; senawi@squ.edu.om; 3 Obstetrics and Gynecology Department, Khoula Hospital, Muscat 113, Oman; rahmaalghabshi@gmail.com; 4 Obstetrics and Gynecology Department, College of Medicine and Hsc, Sultan Qaboos University, Muscat 123, Oman

**Keywords:** recurrent pregnancy loss, infertility, depression, anxiety, stress, psychological morbidity, Omani population

## Abstract

**Highlights:**

**What are the main findings?**

- The cross-sectional nature limits the ability to draw causal inferences.
- The absence of male partner assessments restricts the understanding of the full psychological impact on couples.

**What are the implications of the main findings?**

- This study’s prospective design strengthens the temporal assessment of outcomes.
- The psychological assessments used are validated in Arabic, ensuring the accurate evaluation of mental health in the target population.

**Abstract:**

**Background:** Recurrent pregnancy loss (RPL) and infertility are associated with significant psychological morbidity, including stress, anxiety, and depression. While these impacts are well-documented globally, their prevalence and severity in the Omani population remain unexplored. This study investigates the mental health outcomes of Omani women with RPL and infertility compared to fertile controls. **Objectives:** The objectives of this study are to assess the prevalence of stress, anxiety, and depression in women with recurrent pregnancy loss (RPL) and infertility and compare these rates to women with no fertility concerns in an Omani population. **Design:** This is a prospective, cross-sectional study. **Setting:** This study’s setting consisted of Sultan Qaboos University Hospital and Royal Hospital in Muscat, Oman. **Participants:** This study included 111 women with RPL, 131 women with infertility, and 210 antenatal controls with no fertility issues. **Interventions:** No clinical interventions were administered as this was an observational study. Participants completed validated psychological assessments (DASS-42 and BDI-II). **Primary and secondary outcome measures:** Primary outcomes were the prevalence rates of stress, anxiety, and depression assessed using DASS-42 and BDI-II. Secondary outcomes included sociodemographic correlates and risk factors **Results:** This study included 111 women in the RPL group, 131 in the infertility group, and 210 controls. Among RPL patients, 31% reported stress, ranging from mild to extremely severe, while 35.9% of infertility patients reported stress, compared to 17.1% in the control group (*p* = 0.003). Anxiety was present in 45% of RPL patients, 45.5% of infertility patients, and 28.1% of controls (*p* = 0.019). Depression, measured by DASS-42, was the most prevalent in the RPL group (34.2%), followed by the infertility group (33.6%) and controls (13.8%) (*p* < 0.001). Similar results were observed with BDI-II, with depression rates of 23.4% in the RPL group, 19.1% in the infertility group, and 7.6% in controls (*p* = 0.02). **Conclusions:** Women with RPL and infertility in Oman experience significantly higher levels of stress, anxiety, and depression compared to women without fertility concerns. This study did not assess the mental health of male partners, highlighting the need for further research on the psychological impact on both partners. Future studies should focus on developing psychological support interventions and evaluating their impact on patient outcomes.

## 1. Introduction

Miscarriage, the most common pregnancy complication defined as pregnancy loss before fetal viability [1], affects 15–25% of pregnancies, with prevalence increasing with advanced maternal age [2]. Recurrent pregnancy loss (RPL), defined by the European Society of Human Reproduction and Embryology (ESHRE) and the American Society of Reproductive Medicine (ASRM) as the consecutive loss of two or more pregnancies [1], impacts 3–5% of women globally and 0.8% in Oman [3]. RPL poses significant challenges, leading to higher levels of depression and anxiety and reduced self-esteem in affected couples [4]. Approximately 50% of women who experience miscarriage report psychological morbidity, including elevated anxiety and depressive symptoms, with 10–50% developing major depressive disorder [5]. Kolte et al. reported that 8.6% of women with RPL experience moderate to severe depression, and 41.2% report high stress levels [6].

Infertility, defined by the World Health Organization (WHO) as the failure to achieve pregnancy after 12 months of regular unprotected intercourse [7], affects 10–15% of couples globally. It is a major life event with significant physical and emotional impacts, often causing turmoil, frustration, depression, anxiety, hopelessness, guilt, and feelings of worthlessness [8]. The prevalence of depression in infertile couples ranges from 15 to 58%, with 8–28% experiencing elevated anxiety levels [9].

Despite extensive research globally, the psychological impact of RPL and infertility in the Omani population remains unexplored. This study aims to evaluate the prevalence and severity of stress, anxiety, and depression in Omani women with RPL and infertility, comparing them to fertile controls.

## 2. Materials and Methods

### 2.1. Participants

This was a prospective, cross-sectional study of women attending the RPL and infertility clinics at Sultan Qaboos University Hospital (SQUH) and Royal Hospital, Muscat, Oman, who were invited to participate. The RPL group included patients with ≥2 consecutive miscarriages, and the infertility group included women unable to conceive after 12 months of regular unprotected intercourse, excluding women with secondary infertility with recurrent pregnancy loss. Between September 2018 and July 2021, 111 women with RPL, 131 with infertility, and 210 controls (from antenatal clinics) were recruited. Women attending high-risk pregnancy clinics or antenatal clinics with prior RPL or infertility were excluded from the control group. Additional exclusions included non-Omani women and those with incomplete sociodemographic data or questionnaires.

### 2.2. Sample Size Calculation

The sample size was calculated to compare psychological outcomes between women with fertility-related conditions (recurrent pregnancy loss and infertility combined as a single study group) and antenatal controls, using a 1:1 allocation ratio. Based on an expected prevalence of anxiety (40%) and depression (10%) in the study group, and lower prevalence in controls, a total sample size of 418 participants (209 per group) was required to achieve 80% power at a 5% significance level. The study group was subsequently stratified into two subgroups—women with recurrent pregnancy loss and women with infertility—for subgroup analyses. Ultimately, 242 women were recruited into the study group (111 with recurrent pregnancy loss and 131 with infertility) and 210 women into the control group, which satisfied the overall sample size requirement.

### 2.3. Data Collection

Ethical approval was obtained from institutional research and ethics committees at SQUH and Royal Hospital. Informed written consent was obtained from participants, who were provided with detailed explanations and written study information. Participants completed a paper-based survey covering sociodemographic and psychological assessments (BDI-II and DASS-42). These validated Arabic-language scales were administered to clinic attendees. Additional data, including demographic details, medical and obstetric history, and fertility treatments, were collected from electronic medical records. Participants with missing data were contacted by phone for completion. All data were treated confidentially, stored in an Excel sheet, and accessed solely by investigators.

#### Questionnaire Interpretation

DASS-42 assessed the levels of stress, anxiety, and depression, while BDI-II specifically evaluated depression. Interpretation guidelines for both scales are shown in the Table 1.

### 2.4. Data Entry and Statistical Analysis

Data were entered into EpiData Entry Client version 4.4.2.0. Statistical analyses were performed using SPSS version 28.0.0.0. A Chi square test and Student’s *t* test were used, and a *p*-value of <0.05 was considered statistically significant.

## 3. Results

### 3.1. Study Population Demographics

This study compared the demographic characteristics, psychological distress (DASS-42), and depressive symptoms (BDI-II) between recurrent pregnancy loss (RPL) patients (*n* = 111) and a control group (*n* = 210). The median age was slightly higher in the RPL group (33 years) compared to the control group (28 years), but this was not statistically significant (*p* = 0.09). RPL patients had fewer living children (median: 1 vs. 2, *p* = 0.0007) and eldest children (median: 5 vs. 3 years, *p* < 0.001). There were no significant differences in education or employment status. However, the RPL group had lower monthly income levels (*p* = 0.02). They were also married for longer periods (median: 9 vs. 6.5 years, *p* = 0.05) and reported higher rates of previous miscarriages (first miscarriage: *p* < 0.001; second miscarriage: *p* < 0.001). In terms of psychological distress, the RPL group scored significantly higher on the stress, anxiety, and depression subscales of DASS-42, as well as on BDI-II (all *p*-values <0.001). Overall, the findings suggest that RPL patients experience greater psychological distress and depressive symptoms compared to the control group (Table 2).

This study compared demographic characteristics, psychological distress (DASS-42), and depressive symptoms (BDI-II) between infertility patients (*n* = 131) and a control group (*n* = 210). The median age was similar between the groups (33 vs. 28 years, *p* = 0.09). Infertility patients had significantly fewer living children (median: 0 vs. 2, *p* < 0.001) and eldest children (median: 7 vs. 3 years, *p* < 0.001). There were no significant differences in education levels. However, infertility patients were less likely to be employed (39.7% vs. 50.9%, *p* = 0.04). No significant differences were observed in monthly income. Infertility patients were married for longer (median: 9 vs. 6.5 years, *p* = 0.01) and were more likely to be in polygamous marriages (6.9% vs. 0.5%, *p* = 0.001). They also reported lower parity (median: 0 vs. 2, *p* < 0.001). In terms of psychological distress, infertility patients scored significantly higher on the stress, anxiety, and depression subscales of DASS-42, as well as on BDI-II (all *p*-values <0.001). These findings indicate that infertility patients experience greater psychological distress and depressive symptoms compared to the control group (Table 3).

### 3.2. Prevalence of Stress, Anxiety, and Depression

A multivariate binary logistic regression model was used to identify the independent predictors of psychological distress as measured by DASS-42. The overall model demonstrated modest explanatory power (Nagelkerke R^2^ = 0.095) with a percentage accuracy in classification (PAC) of 75.9%.

After adjusting for all covariates, no variable emerged as a statistically significant independent predictor of psychological distress (*p* > 0.05 for all variables).

Participants in the RPL group had 2.56 times higher odds of psychological distress compared to controls (aOR = 2.557; 95% CI: 0.971–6.738; *p* = 0.057). Similarly, those in the infertility group had 2.40 times higher odds of psychological distress (aOR = 2.400; 95% CI: 0.963–5.982; *p* = 0.060). Although these associations did not reach statistical significance, they indicate a borderline trend toward increased psychological distress in these groups.

None of the sociodemographic variables, including age, educational level, employment status, monthly income, years of marriage, parity, number of miscarriages, number of living children, age of the eldest child, second-wife status, or family psychiatric history, showed a significant association with psychological distress.

Overall, while women with RPL and infertility tended to have higher odds of psychological distress, the multivariate model did not identify any statistically significant independent predictors (Table 4).

Similarly, a multivariate binary logistic regression model was used to identify independent predictors of psychological distress as measured by BDI-II. The overall model showed modest explanatory power (Nagelkerke R^2^ = 0.060) with a percentage of accurate classification (PAC) of 69.6%.

After adjusting for all covariates in the model, none of the studied variables were found to be statistically significant independent predictors of psychological distress (*p* > 0.05).

Age was not significantly associated with psychological distress (aOR = 0.953; 95% CI: 0.885–1.027; *p* = 0.209). Compared to the control group, participants with recurrent pregnancy loss (RPL) (aOR = 1.412; 95% CI: 0.564–3.536; *p* = 0.461) and infertility (aOR = 1.721; 95% CI: 0.727–4.077; *p* = 0.217) did not show significantly higher odds of distress.

Educational level was not associated with psychological distress. Employment status showed a trend toward lower odds of distress among unemployed women (aOR = 0.567; 95% CI: 0.292–1.098; *p* = 0.093), although this did not reach statistical significance. Similarly, lower monthly income (<500 OMR: aOR = 2.041, 95% CI: 0.799–5.216, *p* = 0.136; 500–1000 OMR: aOR = 1.495, 95% CI: 0.755–2.960, *p* = 0.248) was not significantly associated with distress.

Reproductive and obstetric factors, including years of marriage, parity, number of miscarriages, number of living children, age of the eldest child, and second-wife status, were also not significantly associated with psychological distress.

Participants with a family history of psychiatric illness had higher odds of distress (aOR = 3.504; 95% CI: 0.541–22.674; *p* = 0.188), but this association was not statistically significant.

Overall, the findings indicate that none of the examined sociodemographic, reproductive, or clinical variables independently predicted psychological distress in this study population (Table 5). The correlation of psychological distress (DASS-42) and depressive symptoms (BDI-II) was evaluated among infertility patients (*n* = 131) and a control group (*n* = 210). Overall, infertility patients exhibited higher scores on DASS-42, indicating greater psychological distress.

The adjusted odds ratio for the anxiety scale was 2.56 for women with recurrent pregnancy loss and 2.4 for women with infertility. Similarly, the odds ratio for depression in women with recurrent pregnancy loss was 1.41 and 1.72 in women with infertility.

## 4. Discussion

Principal Findings: This study assessed the prevalence of stress, anxiety, and depression among Omani women with recurrent pregnancy loss (RPL) and infertility and antenatal controls without fertility concerns. The prevalence of psychological distress was significantly higher in the RPL and infertility groups compared to controls. Among RPL patients, 31.5% experienced stress, 45.0% anxiety, and 34.2% depression (DASS-42), while in the infertility group, 35.9% reported stress, 45.5% anxiety, and 33.6% depression. The control group demonstrated lower rates of psychological morbidity: stress at 17.1%, anxiety at 28.1%, and depression at 13.8%.

Results in the Context of the Existing Literature: The prevalence of stress in RPL patients (31.5%) was lower than that in Kolte et al.’s findings (41.2%) [6], whereas anxiety (45.0%) and depression (34.2%) exceeded the rates reported by He et al. (7% anxiety) and Wang et al. (28.7% anxiety; 10.8% depression) [10,11]. Among infertility patients, psychological morbidity rates were lower compared to the findings by Yusuf et al. (69–79%), Patel et al. (80% infertility-specific stress), Maroufizadeh et al. (49.6% anxiety, 33.0% depression), Omani-Samani et al. (57% depression), and Haririan et al. (58% depression) [12,13,14,15,16]. The observed variations may reflect differences in study populations, diagnostic tools, and cultural or social contexts [15]. In the control group, the prevalence rates of stress, anxiety, and depression were comparable to or slightly lower than regional studies, such as those by Alqahtani et al. and Al-Azri et al. [17,18].

Clinical Implications: Women with RPL and infertility had slightly higher odds for developing depression and anxiety, but it did not reach statistical significance. Understanding protective factors such as higher education, employment, and having a living child can guide clinicians in identifying women at lower risk. These may be observational associations and not causal. Additionally, tailored interventions for high-risk groups, such as those with recent miscarriages or without fertility treatments, are essential for improving psychological outcomes.

Research Implications: Future studies should explore culturally specific factors that influence psychological resilience and distress in Omani women. Social stability and strong relationships are protective factors against depression and may vary significantly across cultures [19]. Understanding how women in Oman regulate their emotions and cope with stress can help develop mental health programs that align with their cultural values [20]. Additionally, the way depression is expressed differs among cultures, with non-Western populations often emphasizing physical symptoms like fatigue or body pain over emotional ones. Recognizing these unique expressions is essential for accurate diagnosis and effective treatment in the Omani context [21]. Research should also address stigma surrounding mental health in this population, which may prevent women from seeking support, and examine strategies to reduce its impact. These insights can guide the development of culturally sensitive psychological interventions and support systems tailored to the needs of women with RPL and infertility.

## 5. Conclusions

Women with RPL and infertility in Oman experience significantly higher levels of stress, anxiety, and depression compared to antenatal controls, underscoring the need for psychological support tailored to cultural contexts. The multivariate analysis for both groups did not identify any independent risk factors. Addressing stigma and understanding the cultural expressions of distress are critical for developing effective mental health interventions.

## Figures and Tables

**Table 1:** Interpretation of DASS-42.

	Normal	Mid	Moderate		Severe	Extremely Severe
Stress	0–14	15–18	19–25		26–33	≥34
Anxiety	0–7	8–9	10–14		15–19	≥20
Depression	0–9	10–13	14–20		21–27	≥28
**Interpretation of BDI-II**						
	**Normal**	**Mild mood** **disturbance**	**Borderline** **clinical depression**	**Moderate** **depression**	**Severe** **depression**	**Extreme** **depression**
	0–10	11–16	17–20	21–30	31–40	>40

**Table 2:** Demography, DASS-42 and BDI-II in recurrent pregnancy loss (RPL) patients [*n* = 111] versus control group [*n* = 210].

	RPL Group	Control Group	*p*-Value
Age (years), median (IQR)	33 (29; 38)	28 (31; 36)	0.09 ^$^
Living children (numbers), median (IQR)	1 (0; 2)	2 (1; 3)	0.0007
Age of Last Child (years), median (IQR)	5 (4; 7)	3 (2;5)	<0.001
Education, *n* (%)			
Illiterate	0 (0)	1 (0.45)	0.28 ^#^
Reads and writes	0 (0)	2 (0.9)	
Primary school	1 (0.9)	0 (0)	
Preparatory school	4 (3.6)	5 (2.3)	
Secondary school	44 (39.6)	66 (31.4)	
Higher education	62 (55.8)	136 (64.7)	
Employment, *n* (%)			
Employed	51 (45.9)	107 (50.9)	0.39 ^#^
Not employed	60 (54.1)	103 (49.1)	
Monthly income in Omani rials, *n* (%)			
<500	15 (13.5)	45 (21.4)	0.02 ^#^
500–1000	67 (60.4)	94 (44.7)	
>1000	29 (26.1)	71 (33.8)	
Years of marriage (years), median (IQR)	9 (5; 12)	6.5 (3.75; 10)	0.05
Second wife, *n* (%)			
Yes	2 (1.8)	1 (0.5)	0.25 ^#^
No	109 (98.2)	207 (99.5)	
Parity (numbers), median (IQR)	1 (0; 2)	2 (1; 3)	0.0004 ^$^
1st miscarriage (numbers), median (IQR)	3 (3; 5)	0 (0; 1)	<0.001 ^$^
2nd miscarriage (numbers), median (IQR)	0 (0; 1)	0 (0; 0)	<0.001 ^$^
Family psychiatric history			
Nil	111 (100)	203 (96.6)	
Depression	0 (0)	7 (3.4)	
Anxiety	0 (0)	0 (0)	
DASS-42			
Stress, median (IQR)	9 (3; 19)	6 (2; 11)	0.0001 ^$^
Anxiety, median (IQR)	5 (1: 12)	3 (1; 8.25)	0.0001 ^$^
Depression, median (IQR)	5 (1; 12)	2 (0; 6)	<0.001 ^$^
BDI-II, median (IQR)	8 (4; 16)	6 (2; 11)	<0.001 ^$^

Key: DASS-42—Depression Anxiety Stress Scale; BDI-II—Beck Depression Inventory-II; IQR—Interquartile range; *p*-value: ^#^ Chi square test; ^$^ Mann–Whitney U test.

**Table 3:** Demography, DASS-42 and BDI-II in infertility patients [*n* = 131] versus controls [*n* = 210].

	Infertility	Controls	*p*-Value
Age (years), median (IQR)	33 (29; 38)	28 (31; 36)	0.09
Living children (numbers), median (IQR)	0 (0; 1)	2 (1; 3)	<0.001
Age of eldest child (years), median (IQR)	7 (5; 9)	3(2;5)	<0.001
Education, *n* (%)			
Illiterate	0 (0)	1 (0.45)	0.2
Reads and writes	0 (0)	2 (0.9)	
Primary school	0 (0)	0 (0)	
Preparatory school	5 (3.9)	5 (2.3)	
Secondary school	54 (41.2)	66 (31.4)	
Higher education	72 (54.9)	136 (64.7)	
Employment, *n* (%)			
Employed	52 (39.7)	107 (50.9)	0.04 ^#^
Not employed	79 (60.3)	103 (49.1)	
Monthly income in rials, *n* (%)			
<500	19 (14.2)	45 (21.4)	0.22
500–1000	68 (51.9)	94 (44.7)	
>1000	44 (33.9)	71 (33.8)	
Years of marriage (years), median (IQR)	9 (5; 12)	6.5 (3.75; 10)	0.01
Second wife, *n* (%)			
Yes	8 (6.9)	1 (0.5)	0.001 ^#^
No	122 (93.1)	207 (99.5)	
Parity (numbers), median (IQR)	0 (0; 1)	2 (1; 3)	<0.001 ^#^
1st miscarriage (numbers), median (IQR)	0 (0; 1)	0 (0; 1)	0.2
2nd miscarriage (numbers), median (IQR)	0 (0; 0)	0 (0; 0)	0.2
Family psychiatric history			
Nil	111 (100)	203 (96.6)	0.04
Depression	0 (0)	7 (3.4)	
Anxiety	0 (0)	0 (0)	
DASS-42			
Stress, median (IQR)	11 (5; 18)	6 (2; 11)	<0.001 ^$^
Anxiety, median (IQR)	7 (4; 13)	3 (1; 8.25)	<0.001 ^$^
Depression, median (IQR)	(2; 13)	2 (0; 6)	<0.001 ^$^
BDI-II median (IQR)	7 (3; 14)	6 (2; 11)	<0.001 ^$^

Key: DASS-42—Depression Anxiety Stress Scale; BDI-II—Beck Depression Inventory-II; IQR—Interquartile range; *p*-value: ^#^—Chi square test; ^$^—Mann–Whitney U test.

**Table 4:** Multivariate binary logistic regression analysis to determine the independent predictors of psychological distress (DASS-42—Depression Anxiety Stress Scale).

Variable	β	*p*-Value	Adjusted Odds Ratio (aOR)	95% CI for aOR	
				Lower	Upper
Age	−0.028	0.508	0.973	0.896	1.056
Group					
Control (Reference)			1.000		
RPL	0.939	0.057	2.557	0.971	6.738
Infertility	0.875	0.060	2.400	0.963	5.982
Educational Level					
Higher education (Reference)			1.000		
Illiterate/Read write	Not estimated				
Preparatory/Primary school	0.363	0.657	1.438	0.289	7.157
Secondary school	−0.019	0.960	0.982	0.471	2.047
Employment					
Yes (Reference)			1.000		
No	−0.477	0.201	0.621	0.299	1.290
Monthly Income					
>1000 OMR (Reference)			1.000		
<500 OMR	−0.377	0.515	0.686	0.221	2.133
500–1000	0.150	0.684	1.162	0.564	2.394
Years of Marriage	0.006	0.904	1.006	0.906	1.118
Second Wife					
No (Reference)			1.000		
Yes	0.261	0.784	1.299	0.200	8.425
Parity	−0.168	0.642	0.846	0.417	1.715
Number of 1st-Trimester Miscarriages	0.069	0.508	1.072	0.873	1.316
Number of 2nd-Trimester Miscarriages	−0.021	0.908	0.979	0.679	1.410
Number of Living Children	0.274	0.481	1.315	0.614	2.815
Age of Eldest Child	−0.056	0.378	0.945	0.835	1.071
Family Psychiatric Hx					
No (Reference)			1.000		
Yes	0.821	0.403	2.273	0.331	15.588

PAC—75.9%; Nagelkerke R Square—0.095. Women with RPL and infertility tended to have higher odds of psychological distress but no statistically significant independent predictors.

**Table 5:** Multivariate binary logistic regression analysis to determine the independent predictors of psychological distress (BDI-II—Beck Depression Inventory-II).

Variable	β	*p*-Value	Adjusted Odds Ratio (aOR)	95% CI for aOR	
				Lower	Upper
Group	−0.048	0.209	0.953	0.885	1.027
Control (Reference)			1.000		
RPL	0.345	0.461	1.412	0.564	3.536
Infertility	0.543	0.217	1.721	0.727	4.077
Educational Level					
Higher education (Reference)			1.000		
Illiterate/Read write	Not estimated				
Preparatory/Primary school	0.084	0.910	1.087	0.254	4.662
Secondary school	0.117	0.725	1.124	0.585	2.161
Employment					
Yes (Reference)			1.000		
No	−0.568	0.093	0.567	0.292	1.098
Monthly Income					
>1000 OMR (Reference)			1.000		
<500 OMR	0.714	0.136	2.041	0.799	5.216
500–1000	0.402	0.248	1.495	0.755	2.960
Years of Marriage	−0.005	0.918	0.995	0.901	1.098
Second Wife					
No (Reference)			1.000		
Yes	0.158	0.867	1.172	0.183	7.502
Parity	0.211	0.503	1.235	0.666	2.289
Number of 1st-Trimester Miscarriages	0.090	0.365	1.095	0.900	1.331
Number of 2nd-Trimester Miscarriages	−0.015	0.931	0.985	0.700	1.386
Number of Living Children	−0.114	0.740	0.892	0.455	1.750
Age of Eldest Child	−0.030	0.615	0.970	0.863	1.091
Family Psychiatric Hx					
No (Reference)			1.000		
Yes	1.254	0.188	3.504	0.541	22.674

PAC—69.6%; Nagelkerke R Square—0.060. None of the studied variables were found to be statistically significant independent predictors of psychological distress.

## Data Availability

The datasets used during the current study are available from the corresponding author on reasonable request.

## Acronyms

ART	Assisted Reproductive Technology
ASRM	American Society for Reproductive Medicine
aOR	Adjusted Odds Ratio
BDI-II	Beck Depression Inventory-II
CBD	Case-Based Discussion
CI	Confidence Interval
DASS-42	Depression Anxiety Stress Scale-42
ESHRE	European Society of Human Reproduction and Embryology
IVF	In Vitro Fertilization
OMR	Omani Rial
PAC	Percentage Accuracy in Classification
RPL	Recurrent Pregnancy Loss
SMJ	*Saudi Medical Journal*
SPSS	Statistical Package for the Social Sciences
SQUH	Sultan Qaboos University Hospital
WHO	World Health Organization
